# Editorial comment: Cross-cultural adaptation and validation of the neurogenic bladder symptom score questionnaire for brazilian portuguese

**DOI:** 10.1590/S1677-5538.IBJU.2018.0335.1

**Published:** 2019-07-27

**Authors:** Blayne Welk

**Affiliations:** 1 Division of Urology and Epidemiology and Biostatistics, Western University, London, UK

Twenty-firstst century medicine emphasizes a patient centered approach with shared decision making. As part of this process, being aware of the patient's quality of life (QoL) is essential when making decisions about their medical care. However, QoL is an abstract concept which is difficult to measure objectively. Just as tools such as urodynamics are developed to measure physiological parameters in the human body, patient reported outcome measures (PROM) are developed to quantify abstract concepts such as QoL and symptom burden. These tools allow researchers to measure the patient's perception of their QoL or symptom burden. Specialized PROMs are often developed to be able to assess a very specific aspect of a patient's life, and ideally they undergo a rigorous qualitative and quantitive process to ensure they are valid.

Cintra and colleagues (1) have reported on the cross-cultural translation and validity of the Neurogenic Bladder Symptom Score (NBSS). This is an important, and sometimes overlooked step to ensure a PROM is useable outside of their original language and patient population. This process takes into account differences in societal norms, socioeconomic status, linguistic context, and medical management. As an example, in North America the expression “feeling blue” refers to depression, whereas in some Latin American cultures this is used as an expression of joy. At the completion of this process, a PROM would be considered equivalent to the original.

The authors should be commended for undertaking this process in accordance with the accepted methods for cross-cultural adaption, and their contribution includes another independent assessment of the validity and reliability of the NBSS. Research in neuro-urology requires the use of high-quality instruments, and this also applies to PROMs. Just as BPH symptom scores can easily communicate a patient's symptom burden, or be used as evidence of efficacy in the regulatory approval of a new drug, the NBSS can be used to measure patients symptoms, their change over time, and quantify and compare the improvement after different procedures. As the developer of the NBSS, my obvious bias is to see this tool used in clinical practice and in research, and I hope the work of Dr. Gomes team will allow the NBSS to play a role in the care of neurourology patients in Brazil.
